# Impact of muscle fatigue on anticipatory postural adjustments during gait initiation

**DOI:** 10.3389/fphys.2024.1520578

**Published:** 2025-01-13

**Authors:** Jorge L. Storniolo, Veronica Farinelli, Roberto Esposti, Paolo Cavallari

**Affiliations:** 1 Laboratorio Sperimentale di Fisiopatologia Neuromotoria, IRCCS Istituto Auxologico Italiano, Meda, Italy; 2 Human Physiology Section of the Department of Pathophysiology and Transplantation, Università degli Studi di Milano, Milan, Italy

**Keywords:** postural strategies, APAs, trunk muscles, exhausting exercise, human

## Abstract

**Introduction:**

Prolonged or strenuous exercise leads to a temporary decrease in muscle function and performance, which interferes with activity of both prime movers and postural muscles. This effect of fatigue has been reported both for single segment movements and for locomotion. However, little is known regarding the effects of fatigue on anticipatory postural adjustments (APAs) during gait initiation, a task in which the control of focal movement should be strictly coupled to a feedforward control of posture.

**Methods:**

We studied APAs during gait initiation in 16 healthy well-trained adult males, searching for muscle activities that precede the backward shift of the Center of Pressure (CoP). Participants stood on a force plate for about 10 s and then started walking at their natural speed. APAs were evaluated before and after a 1 min exhausting sequence of countermovement jumps. An optoelectronic system captured the heel-off events while a force plate measured the CoP position and vertical ground reaction force. Wireless probes recorded the electromyogram of trunk and leg muscles from both sides.

**Results:**

It was observed that muscle fatigue delayed excitatory and inhibitory APAs, of about 40 and 80 ms, respectively, and a parallel delay was induced on prime movers; moreover, velocity and amplitude of backward CoP shift were reduced. Regarding APAs sign and occurrence, most of the participants showed bilateral inhibition in dorsal muscles and excitation in the ventral ones, displaying a forward “diving” strategy that was almost unaffected by fatigue. However, after fatigue, three of the “diving” participants switched to a “turning” strategy, i.e., they displayed a reciprocal activation/inhibition pattern in the dorsal muscles, compatible with a trunk rotation.

**Discussion:**

The “turning” strategy has been previously described in untrained individuals and in a toes-amputee mountain climber, who showed a “diving” approach to gait initiation when wearing his prosthetic shoes and switched to the “turning” approach when barefoot. Altogether, these results support the idea that one and the same person may develop a repertoire of postural strategies among which the central nervous system will choose, according to the personal fitness and the constraints in which the action is performed.

## 1 Introduction

Physiological fatigue is a complex process involving various factors leading to a decline in muscle performance (Bigland-Ritchie and Woods, 1984). Fatigue, characterized by reduced maximal muscle force or power due to prolonged or strenuous exercise, may result from peripheral mechanisms including metabolic by-products, depletion of energy substrates, accumulation of extracellular potassium ions, and alterations in neuromuscular function (Davies et al., 1991). Additionally, also central fatigue, stemming from changes in the central nervous system (spinal motor neurons, motor cortex, or structures upstream of the motor cortex), has been shown to contribute to tiredness and reduced muscle output, leading to twitching, cramps, aches, and pains (Gandevia, 2001). These processes may interact and influence each other, leading to a temporary decrease in muscle function and performance (for a recent review, see Behrens et al., 2023).

Actually, it has been demonstrated that the increase in blood lactate levels after fatigue not only impairs cognitive and executive functions, but also exert direct cortical influences by increasing primary motor cortex excitability and depressing supplementary motor area (Coco et al., 2016; 2020). In turn, Bolzoni et al. (2015) showed that changes in supplementary motor area excitability modulates Anticipatory Postural Adjustments (APAs) without affecting the primary movement. Taken together, the above cited results support the idea that the fatigue effects may also involve the postural control, in particular the APAs that stabilize posture prior to the execution of the focal movement.

A few studies reported that fatigue impairs postural control and locomotion in young adults and older participants; actually, increased occurrence of falls is not only caused by biological aging, but also by fatigue in lower leg muscles (Helbostad et al., 2007; Parijat and Lockhart, 2008). Specifically, in healthy young adults it was observed that localized muscle fatigue of the quadriceps affected various kinematic and kinetic parameters linked with a high risk of slip-induced falls (Parijat and Lockhart, 2008). In parallel, a repeated sit-to-stand task impaired locomotion control in older persons with regard to increased step width and length variability (Helbostad et al., 2007).

The intrinsic complexity of walking becomes apparent at its very beginning, i.e., at gait initiation, when the control of focal movement should be strictly coupled to a feedforward control of posture. Indeed, starting gait implies not only to propel the Center of Gravity (CoG), but also to build-up the postural scheme needed to grant body stability in the transition from quiet standing to locomotion (Farinelli et al., 2021). In this framework, fatigue should likely affect not only the movement itself but also the postural actions preceding it. Actually, a conclusion in this direction is reported by Yiou et al. (2011), who showed an increased duration of the APAs after acute fatigue of Tibialis Anterior (TA) muscles. This prolonged activity was however expressed as the time difference between the onset of forward acceleration of the CoG and the take-off of the leading foot. In parallel, TA activation was depressed, while its onset remained locked to that of CoG acceleration. From a functional point of view, decreased electrical activity during gait initiation in both TAs may reflect a protective strategy, aiming to attenuate the intensity of the contraction and thus optimally preserve the integrity of the fatigued muscle. In turn, such result is in agreement with the “pain-adaptation model” (Lund et al., 1991), which predicts a decrease in EMG activity of agonist muscles, an increase in EMG activity of antagonist muscles, and slower and less powerful movements during muscle pain to protect the painful muscle.

However, such results are not exhaustive for the following reasons: i) the TA and its antagonist Soleus (Sol) should be considered prime movers, not postural muscles, since they shift the Center of Pressure (CoP) backward and consequently propel the CoG forward; ii) the forward CoG acceleration and the ensuing take-off of the leading foot are intrinsic part of the focal movement, thus the APAs should be searched for before those events. In this perspective, the APAs associated with gait initiation has been detailed by Farinelli et al. (2021); in this paper we address the question on whether APAs associated with gait initiation may be affected by whole-body fatigue.

## 2 Methods

### 2.1 Participants

Seeking homogeneity in the effectiveness of the fatigue procedure (see below), the study was conducted on healthy male adults, free from any musculoskeletal or neurological dysfunction and systematically practicing physical activity, so as to control for effects of age, gender and training status. Specifically, there were enrolled sixteen men with an age of 26 ± 6 years (mean ± SD), a height of 1.76 ± 0.1 m, a weight of 75 ± 10.1 kg and practicing physical activity at least three times per week. All participants were right-footed, as ascertained by asking them which leg they used to kick a ball, as well as by observing the limb used to initiate walking spontaneuosly. The experimental procedure was carried out in accordance with the standards laid down in the Declaration of Helsinki and approved by the “Comitato Etico di Ateneo dell'Università degli Studi di Milano” (counsel 23/23).

### 2.2 Experimental setup

**FIGURE 1:**
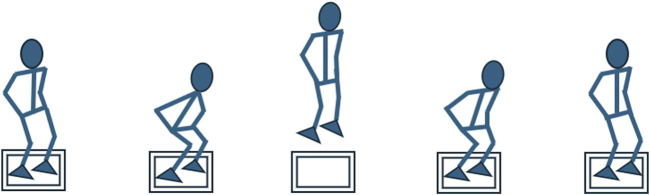
Starting the countermovement jump sequence: 1: standing position; 2: knees flexion to squat; 3: jump and take off from the force plate; 4: landing on the force platform; 5: pushing to jump again.

The experimental session consisted of a gait initiation task. Specifically, participants wearing sports shoes were asked to stand on the force plate for 10 s and then start walking at their own will after a vocal prompt. Ten trials were collected before and after muscle fatigue. The fatiguing procedure consisted of a 1-min of uninterrupted countermovement jumps (CMJ), i.e., a sequence of vertical jumps performed by quickly squatting with the hands on the hips, then jumping as high as possible and repeating the squat-jump sequence immediately after each landing (Bosco et al., 1983; Figure 1). Simultaneously, the participant was verbally encouraged to reinforce the maximal effort required for the test.

### 2.3 Recordings

A dynamometric force plate (9286AA, KISTLER, Winterthur, Switzerland, sampling frequency: 400 Hz) was used to compute the CoP position and measure the vertical Ground Reaction Force (vGRF) while jumping. Wireless probes (FREEEMG 1000, BTS, Milan, Italy, sampling frequency: 1,000 Hz) were employed bilaterally to record the surface electromyographic (EMG) activity of TA, Sol, Obliquus Abdominis (OA), Erector Spinae at L2 vertebra (ES), Vastus Medialis (VM) and Biceps Femoris (BF). Electrodes were placed according to the Surface Electromyography for the Non-Invasive Assessment of Muscles (SENIAM) guidelines (Hermens et al., 2000). Markers on the heels were recorded through an eight-camera optoelectronic system (SMART-DX, BTS, Milan, Italy, sampling frequency 100 Hz), allowing the estimation of the heel-off events.

### 2.4 Data processing

EMG data were processed as described elsewhere (Farinelli et al., 2021). In brief, after high-pass filtering (50 Hz for all muscles except OA, in which 150 Hz was used to reject cardiac artifacts), EMG traces were full-wave rectified, then time-aligned to the heel-off of the leading foot and averaged across trials. The same averaging was applied to CoP and heel traces. The mean level and SD of the CoP trace from 3 to 1 s before the heel-off was measured, then the onset of the backward CoP shift was identified by a custom-made algorithm, which searched for the time point where the trace fell below −2 SD with respect to the mean level and remained below that value for at least 50 ms. CoP onset was then assigned to Time 0. The averaged EMGs were integrated with a 25 ms moving window (the window outcome was assigned to the central sample to avoid phase-lags) and the mean level and SD of the traces were measured from 3 to 1 s before the CoP onset. The onset of the EMG changes was identified by applying the same algorithm used for CoP setting the threshold to mean ±2 SD and restraining the search from −300 to +100 ms with respect to CoP onset. Amplitude and latency of the first backward peak of CoP were also measured.

To assess the effectiveness of the fatigue procedure, the number of jumps and the average of the vGRF peaks in the propulsive phase, i.e., the last peak before flight, were considered. Both variables were calculated in two time windows: in the first and in the last 15 s of the 1-min fatigue procedure.

### 2.5 Statistical analysis

First, the occurrence of the EMG changes was scored by assigning +1 to excitation, −1 to inhibition, and 0 in case of absence; then, considering the categorical nature of such variable, the effect of fatigue was assessed by Wilcoxon matched pairs test. The latency of EMG changes was considered only for those participants who showed changes of the same sign in both conditions. In view of the continuous nature of the variable, the normality was preliminarily checked in each muscle by Shapiro-Wilk test; then the effect of fatigue on APA latency was evaluated either by paired t-tests, for those muscles in which normality was not violated, or by Wilcoxon matched pairs test where the normality assumption did not hold true. The same procedure was used to compare amplitude and latency of backward CoP shift before and after fatigue as well as for evaluating the outcome of the fatiguing procedure, in which the number of jumps and the average peak of the vGRF were compared in the first and last 15 s of exercise. The normality assumption did not hold true only for the latency of BF on the trailing side and of TA on the leading side. Significance threshold was set at p = 0.05.

## 3 Results

### 3.1 Efficacy of the fatiguing protocol

The CMJ procedure did not decrease the jump frequency, indeed the number of jumps performed by the participants was, in average, comparable during the first and last 15 s of the exercise (13.8 ± 0.4 vs. 14.1 ± 0.6; t-test p = 0.48). On the contrary, the explosive force dropped by about 18%: the peak force of each jump being 1,471 ± 41 N (mean ± SE) in the first 15 s and 1,203 ± 40 N in the last 15 s (t-test p < 0.0001).

### 3.2 Impact of muscle fatigue on APAs during gait initiation

Our protocol of muscle fatigue induced visible changes in sign and occurrence of APAs, as well as in their latency.

For what regards sign and occurrence (inhibition, excitation or absence) fatigue induced effects of increased severity from distal to proximal postural muscles. This could be appreciated by examining both the occurrence of excitation/inhibition (left side of Table 1) and the number of those participants who showed EMG changes of the same sign in both conditions (i.e., the matched pairs, right side of Table 1). For example, the inhibition of BF muscle in the trailing side was observed in 12 participants in control condition and in 13 after fatigue. However, only 10 individuals showed inhibition in both conditions. This means that two individuals missed the original inhibition after fatigue while 3 displayed inhibitions only after fatigue. It is then clear that fatigue had no systematic effect, as witnessed by the Wilcoxon test. The most severe effects were observed in the ES of the trailing side and in the OA of the leading side, in which the Wilcoxon test reached significance. Indeed, out of the 7 participants showing excitation in control, 4 dropped it after fatigue; instead, two further individuals displayed inhibition instead of excitation, but only in fatigue.

**TABLE 1:** Effect of fatigue on Signs, Occurrence and Latency of APAs in postural muscles Erector Spinae (ES), Obliquus Abdominis (OA), Biceps Femoris (BF) and Vastus Medialis (VM), as well as on EMG activity of prime movers Soleus (Sol) and Tibialis Anterior (TA). On the left side, number of participants showing inhibition (blue), excitation (red) or absence (black) of APA/EMG activity, compared by Wilcoxon matched pairs test. On the right, the Mean Latency was calculated only for matched pairs (i.e., those participants who showed EMG changes of the same sign in both conditions) and compared either by t-test (^**t**^), where data distribution did not deviate from normality, or by Wilcoxon matched pairs test (^**W**^) where the normality assumption was violated. Significant p-values in bold.

		Sign and occurrence (n/16 participants)			Mean latency ± SE (ms) for matched pairs		
		Control	Fatigue	p	Control	Fatigue	p (n pairs)
Leading side	ES	12	11	>0.5	−158 ± 15	−91 ± 18	**0.011** (11) t
		1	-		-	-	-
		3	5		-	-	-
	OA	-	2	**0.028**	-	-	-
		7	3		−77 ± 26	−43 ± 10	n.c. (3)
		9	11		-	-	-
	BF	9	12	0.225	−173 ± 16	−97 ± 33	**0.020** (8) t
		-	-		-	-	-
		7	4		-	-	-
	VM	-	-	>0.5	-	-	-
		12	11		−105 ± 11	−51 ± 18	**0.009** (11) t
		4	5		-	-	-
	Sol	13	12	>0.5	−123 ± 29	−61 ± 20	**0.007** (12) t
		-	-		-	-	-
		3	4		-	-	-
	TA	-	-	>0.5	-	-	-
		13	14		−79 ± 21	−36 ± 19	**0.046** (13) W
		3	2		-	-	-
Trailing side	ES	11	9	0.176	−139 ± 19	−76 ± 32	**0.037** (7) t
		1	4		−24	39	n.c. (1)
		4	3		-	-	-
	OA	-	-	>0.5	-	-	-
		12	12		−45 ± 12	−5 ± 16	**0.017** (12) t
		4	4		-	-	-
	BF	12	13	>0.5	−167 ± 24	−80 ± 16	**0.005** (10) W
		-	-		-	-	-
		4	3		-	-	-
	VM	-	-	>0.5	-	-	-
		14	14		−69 ± 12	−23 ± 17	**0.011** (13) t
		2	2		-	-	-
	Sol	16	15	>0.5	−147 ± 16	−78 ± 19	**0.003** (15) t
		-	-		-	-	-
		-	1		-	-	-
	TA	-	-	>0.5	-	-	-
		16	15		−78 ± 15	−41 ± 12	**0.04** (15) t
		-	1		-	-	-

The disturbing effect of fatigue was not limited to the EMG pattern (sign and occurrence), but also became apparent in the latency of both excitation and inhibition (right side of Table 1). In fact, all trunk and thigh muscles demonstrated a significant delay in APA activity after fatigue, which was confirmed by statistics. This was also paralleled by the activity of the prime movers (TA recruitment and Sol inhibition). Moreover, it is worth to note that the amount of delay was comparable among muscles showing excitation (∼40 ms) as well as among those showing inhibition (∼80 ms).

**FIGURE 2:**
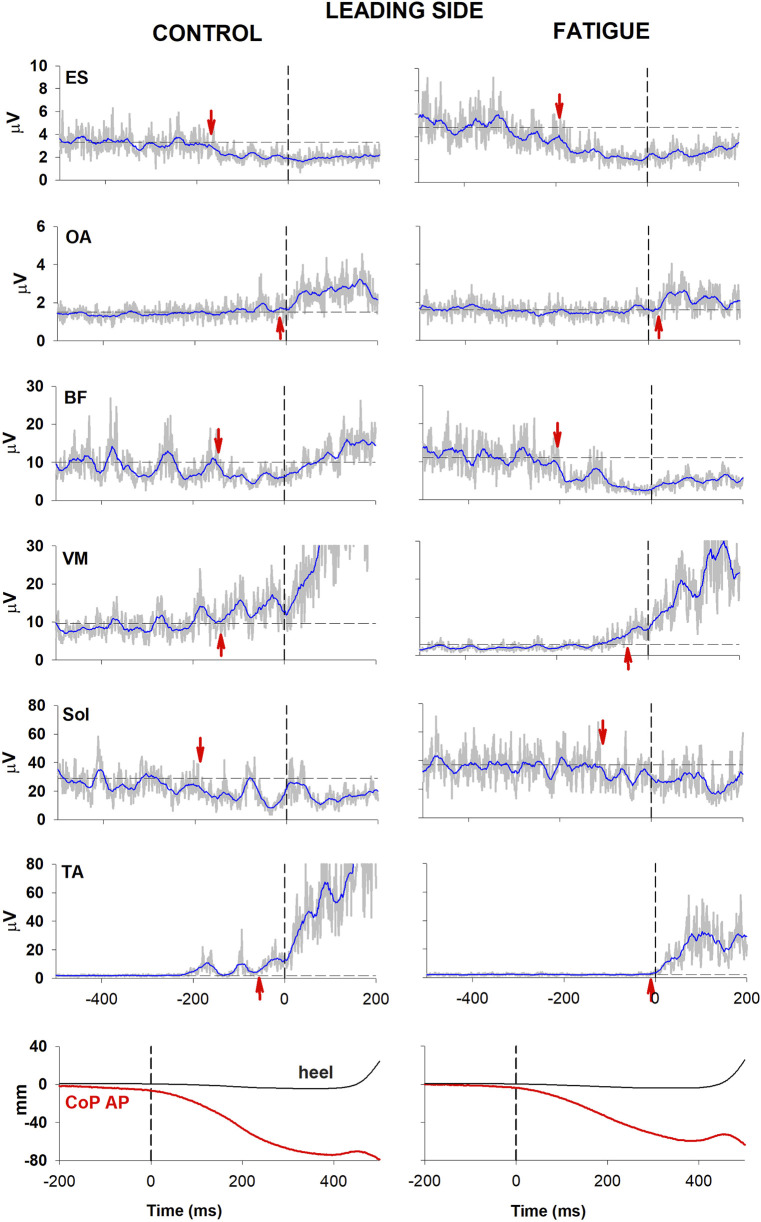
Comparison of raw (grey) and integrated (blue) EMG activity in control (left) and fatigue (right) condition on the leading side. One participant in which all muscular activities kept the same sign before and after fatigue. From the top: Erector Spinae (ES), Obliquus Abdominis (OA), Biceps Femoris (BF), Vastus Medialis (VM) and prime movers Soleus (Sol) and Tibialis Anterior (TA). Arrows mark the onset of excitation (upward arrow) or inhibition (downward arrow). Black dashed lines at time 0 mark the onset of the backward whole-body CoP shift while the horizontal lines represent the average muscle activity at baseline. The lowermost plot shows the anteroposterior CoP displacement (red) as well as the heel vertical displacement (black).

**FIGURE 3:**
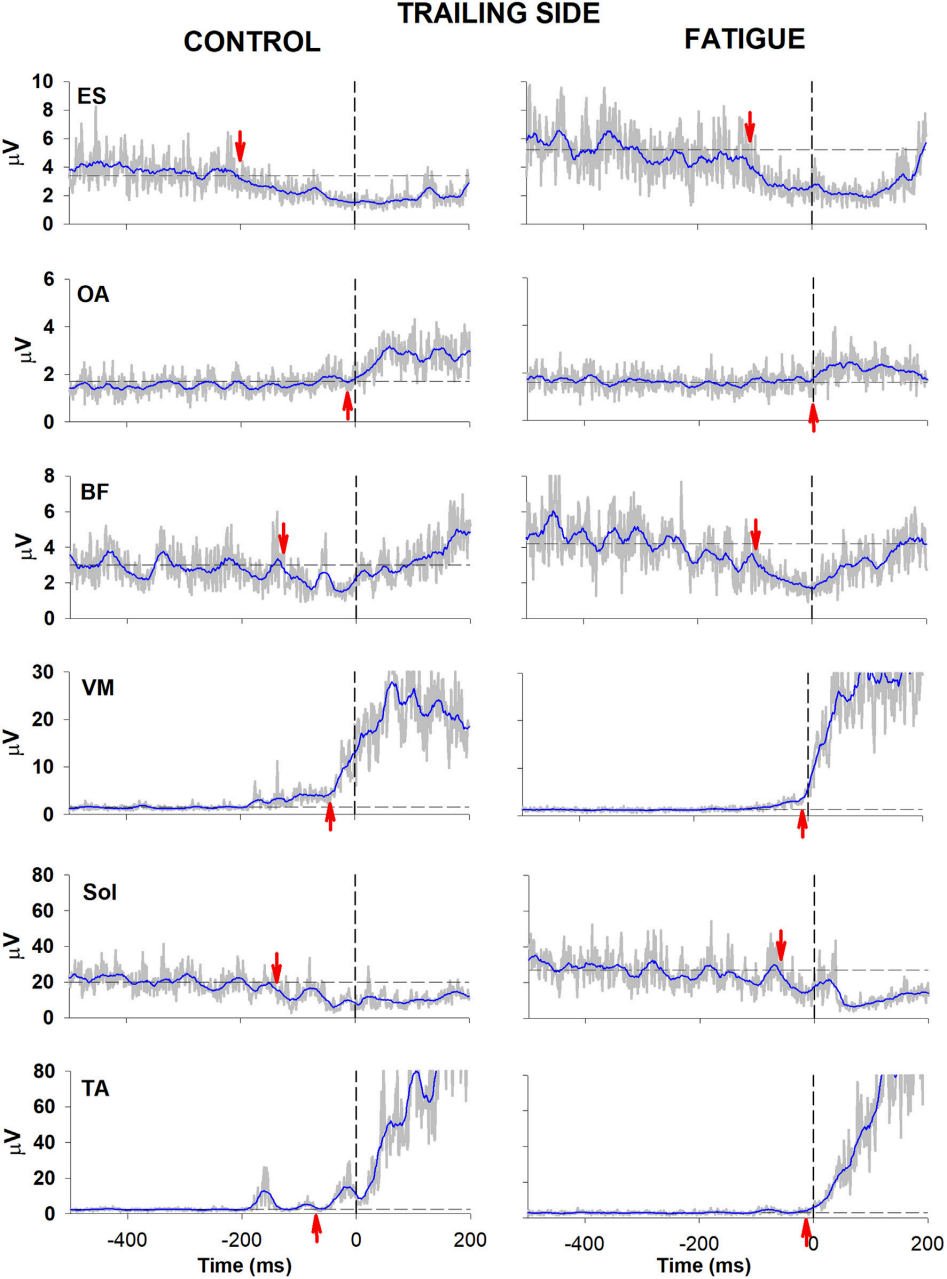
Comparison of raw (grey) and integrated (blue) EMG activity in control (left) and fatigue (right) condition on the trailing side. Same participant and graphic conventions as in Figure 2.

Figures 2, 3 illustrate the behaviour of one participant in which all muscular activities kept the same sign before and after fatigue. In the control condition, EMG changes preceding CoP shift could be identified in the trunk and lower limb muscles of both the trailing and the leading sides (left panel, Figures 2, 3). Specifically, as in most participants, a reciprocal activity was observed in dorsal and ventral postural muscles. Indeed, ES and BF showed an inhibition of tonic EMG activity while OA and VM showed an excitation. These APAs accompanied the well-known reciprocal activity observed in the prime movers (inhibition in Sol and excitation in TA). After the fatiguing procedure (right panel, Figures 2, 3), a clear increase in latency occurred in all muscular activities.

Fatigue also affected the CoP trajectories. Indeed, in the illustrated participant the fatigue procedure reduced the amplitude of the peak backward shift, without changing its latency (bottom traces in Figure 2). This was measured in the whole population, resulting into a significant reduction of peak amplitude (46.55 mm ± 4.91 SE in control condition vs. 40.71 mm ± 4.07 SE in fatigue; t-test p = 0.047) while latency was almost unchanged (449 ms ± 26 vs. 439 ms ± 20; t-test p > 0.5).

## 4 Discussion

### 4.1 Neuromuscular and biomechanical factors affecting APA timing and pattern

With the aim of assessing whether the effect of fatigue alters the APAs and the prime movers activity in physically active participants, our results highlighted that the CMJ procedure mainly delayed APAs and reduced the speed of the backward CoP shift, with minor effects on sign and occurrence of APAs.

There is an apparent problem in comparing our findings with those reported by literature. Indeed, 1) most studies on the effect of fatigue on APAs consider upper limb movements, not gait initiation; 2) only few studies employed a fatigue task affecting both prime movers and postural muscles, like our CMJ task; 3) the definition of the reference time for measuring APA latency is different among papers. Specific references are provided in the following paragraphs.

In regard to APA timing, it should be noted that we employed a novel approach (Farinelli et al., 2021) which reinterpreted the posturo-kinetic chain related to gait initiation. In fact, classical literature searched for APAs in the time window from the first CoP displacement to the onset of heel-off or foot-off (Crenna and Frigo, 1991; Assaiante et al., 2000; Yiou et al., 2011; Yiou et al., 2017), including activities in TA and Sol. This approach neglected the fact that initiating gait requires to push forward the CoG, an action that is attained by shifting the CoP backward. In our view, the CoP displacement is the effect of prime movers, meaning that Sol and TA activity should no longer be considered APAs but rather the expression of the primary motor command. Consequently, APAs should be ideally searched for in a time window preceding the backward CoP displacement, i.e., well before the heel-off. Thus, the timing of our EMG activities is not directly comparable with that measured in previous studies on gait initiation, like in Yiou et al. (2011), who reported an earlier onset of APAs after fatigue. However, we observed a reduction in backward CoP velocity, as described by Yiou.

Our approach is instead much more congruent with that used in classical papers on APAs accompanying movements of single segments (for a review see Bouisset and Do, 2008). In this regard, literature on arm raising movements reports contrasting results. Kanekar et al. (2008) described a minimal APAs anticipation (less than 10 ms with respect to prime mover activation) in ES, BF and Semitendinosus after fatiguing postural muscles. This result is also supported by Monjo and Forestier (2014), who induced muscle fatigue by electrical stimulation, as well as by Schouppe et al. (2019) who studied APAs after physical and cognitive exertion. No changes in APAs after fatigue were also reported by Yiou et al. (2009) during bilateral forward reach task. Conversely, Strang and Berg (2007) described up to 25 ms advance in Paraspinals muscles. Slightly larger advances (about 30–40 ms) were reported by Vuillerme et al. (2002) in the Semitendinosus. Similarly, Allison and Henry (2002) found about 30–40 ms advance in Rectus Abdominis and OA, but only in 4 participants. Our results are in contrast with those of Strang and Berg (2007) and Vuillerme et al. (2002) but in agreement with Kanekar et al. (2008), Monjo and Forestier (2014) and Schouppe et al. (2019). Indeed, we observed that prime movers and postural muscles underwent a comparable time shift both for excitatory and inhibitory actions, witnessing that the time difference between APAs and prime movers onset did not change more than 10 ms. The high variability of outcomes has been also addressed in a review by Yiou et al. (2012) concerning the APA adaptability to biomechanical, physiological, temporal and psychological constraints. In regard to fatigue, a neurophysiological constraint, these authors concluded that its effect on APAs depends on the fatiguing procedure and the mechanical requirements of the motor task.

### 4.2 APA pattern associated with different strategies of gait initiation

Another aspect to discuss is the pattern of APAs in back muscles. When we introduced the new perspective on APAs in gait initiation (Farinelli et al., 2021) we observed that healthy participants systematically adopted a “turning” strategy around the trailing body side, which acted as a pivot. Indeed, the ES and abdominal muscles were excited on the trailing side while, on the leading side, ES was inhibited and abdominals excited.

On the contrary, in the current work, ES APAs were usually inhibitory on the leading and trailing sides, thus promoting a “diving” strategy, both in control and fatigue conditions. There are however a few exceptions: one participant showed reciprocal APAs in ES (i.e., the “turning” strategy) in both conditions and, interestingly, 3 participants developed reciprocal activation of the ES specifically after fatigue. The question then arises as to why there is a different pattern of ES activity not only between participants but also even in one and the same individual. This suggests that different strategies may be available to the Central Nervous System (CNS), which then chooses the most suitable one according to the individual’s needs.

This perspective is sustained by a recent observation (Storniolo et al., 2024) that a change in gait initiation strategy occurred in a very trained individual (professional climber) who undergone bilateral amputation of all five toes. When barefoot, a condition of greater instability due to the amputation, such participant adopted the slower and seemingly safer “turning” strategy, while when wearing prosthetic shoes, which restored the total length of the feet and likely increased the self-confidence, he adopted the “diving” strategy. It should also be noted that participants in Farinelli et al. (2021) were untrained and barefoot people, while in the present paper the participants were well-trained and shod. For what concerns the level of physical activity, here we enrolled trained participants, seeking for homogeneity in the effectiveness of the fatigue procedure. On the other hand, with regard to the barefoot vs. shod condition, we know that donning or doffing shoes may influence the gait initiation strategy (Storniolo et al., 2024). However, shoes were required in the present study to protect lower limb joints from the strong impacts with the ground during the CMJ. It could then be argued that to compare actual data with those of Farinelli et al. (2021) shoes might have been donned only during CMJ. Unfortunately, we have no idea of how long it would take, after doffing shoes, to restore the “barefoot” gait strategy, while we had to speed-up the gait initiation recording before fatigue fading. Thus, we chose to keep the *disturbing* effect of shoes throughout the experimental session, in the aim of evaluating the pure effect of fatigue.

Both of the above differences clearly preclude a direct comparison of present data on gait initiation with those of Farinelli et al. (2021). At the same time, however, the fact that present participants were well-trained and shod likely increased their self-confidence, as it occurred in the amputated climber when shod. It can then be argued that the “diving” strategy may be changed into the “turning” one when self-confidence decreases, a condition that may occur after fatigue. Indeed, it has been observed in rugby players that neuromuscular fatigue induced by the CMJ procedure can manifest itself not only in the reduction of physical production, such as jump height, but mainly as an altered motor strategy (Kennedy and Drake, 2017). This perspective is also in agreement with the conclusion by Yiou et al. (2012) that “depending on the constraints, the priority of the CNS was focused on postural stability maintenance, on body protection and/or on maintenance of focal movement performance”. Therefore, the CNS can adapt strategies according to different conditions. As an example, different strategies for body progression are adopted by toddlers after a few months of independent walking (Bisi and Stagni, 2015): some of them use the “stepper” strategy, with a trunk rotation (similar to our “turning” strategy) while others display a “controlled forward fall” (similar to our “diving” approach). This seems also to occur in elderly people who adopt the “turning” strategy as long as they lose their self-confidence (Onuma et al., 2020). Interestingly enough, Rowland et al. (2021) reported delayed APAs in ES muscles of elderly people performing bilateral arm rising, a situation that can be reconducted to the APA delay observed in our fatigued participants.

In conclusion, present results, combined with those of Farinelli et al. (2021) and Storniolo et al. (2024), support the existence of a repertoire of postural strategies among which the CNS chooses, according to the specific constraints in which the action is to be performed; a view in agreement with that proposed by Yiou et al. (2012). This concept can be also reconducted to Bernstein’s idea that any voluntary movement may be associated with an arborized pattern of postural commands, each tailored to a specific context. Accordingly, an APA pattern may be shaped to fit the mechanical needs and be maintained subliminal until necessary; by amplifying transmission to certain targets and attenuating transmission to others, the APA chain is brought to action wherever needed (Baldissera et al., 2002; Baldissera and Esposti, 2005).

### 4.3 Limitations and future directions

We purposely limited the experimental sample to healthy male adults, well fitted and shod. While this selected sample allowed to rule-out possible confounding factors (see Methods), it would be of interest to enrol a more variate sample and stratify the outcomes according with the different anthropometric characteristics, ages, gender and level of physical activity. In this regard, it would also be of interest to compare athletes practicing disciplines that require different postural approaches.

Another limitation regards the analysis of body kinematics, which we restrained to antero-posterior CoP displacement as such variable has been recently introduced to set the APA time window for the analysis of postural muscles (Farinelli et al., 2021). Future studies could investigate the CoP/CoM displacements in both antero-posterior and medio-lateral directions, with or without fatigue. Such topic has been already addressed (Honeine et al., 2016), but using the classical approach of searching for APAs in the time window from the first CoP displacement to the onset of heel-off, and without applying fatigue.

## Data Availability

The raw data supporting the conclusions of this article will be made available by the authors, without undue reservation.
