# Correction: Impact of muscle fatigue on anticipatory postural adjustments during gait initiation

**DOI:** 10.3389/fphys.2025.1764811

**Published:** 2026-01-13

**Authors:** Jorge L. Storniolo, Veronica Farinelli, Roberto Esposti, Paolo Cavallari

**Affiliations:** 1 Laboratorio Sperimentale di Fisiopatologia Neuromotoria, IRCCS Istituto Auxologico Italiano, Meda, Italy; 2 Human Physiology Section of the Department of Pathophysiology and Transplantation, Università degli Studi di Milano, Milan, Italy

**Keywords:** postural strategies, APAs, trunk muscles, exhausting exercise, human

**Author** Jorge L. Storniolo was erroneously assigned to **Affiliation** Human Physiology Section of the Department of Pathophysiology and Transplantation, Università degli Studi di Milano, Milan, Italy. This **Affiliation** has now been removed for **Author** Jorge L. Storniolo.

The original article has been updated.

